# On the conflict between science and ethics: the case of the moggel, *Labeo umbratus* (Teleostei: Cyprinidae) from South Africa

**DOI:** 10.1111/jfb.70263

**Published:** 2025-11-06

**Authors:** Mpho Ramoejane, Fatah Zarei, Xiluva Mathebula, Albert Chakona

**Affiliations:** ^1^ Department of Zoology and Entomology University of the Free State Phuthaditjhaba South Africa; ^2^ NRF‐South African Institute for Aquatic Biodiversity Makhanda (Grahamstown) South Africa; ^3^ Department of Ichthyology and Fisheries Science Rhodes University Makhanda (Grahamstown) South Africa

**Keywords:** endemic species, Great Fish system, integrative taxonomy, *Labeo umbratus* species‐group, Orange‐Vaal system

## Abstract

Recent molecular evidence showed that *Labeo umbratus* comprises two allopatrically distributed genetic lineages, a northern lineage confined to the Orange‐Vaal River system where this species was originally described and a southern lineage with three geographically separated haplogroups (western, central and eastern) distributed across multiple isolated southward‐draining river systems in the Cape Fold, Amatola‐Winterberg Highlands, and the Southern Temperate Highveld freshwater ecoregions of South Africa. Detailed morphological examination supported recognition of the eastern haplogroup, distributed from the Sundays to the Nahoon River system, as a distinct species. Although a name proposed in 1861 exists for this haplogroup, originally described from the Kat River, a branch of the Great Fish River system, it is derogatory and offensive to the indigenous people of South Africa and has not been adopted in subsequent taxonomic literature. We see no nomenclatural stability in resurrecting a name that is both offensive and long forgotten, and we argue that retaining such a name undermines universality because it conflicts with ethical and cultural acceptance in the region of endemism. Accordingly, we reject the use of this derogatory name and instead provide a new name, *Labeo ngqikai*, for the species to ensure both nomenclatural stability and cultural sensitivity. The epithet *ngqikai* honours King Ngqika, the third paramount chief of the native inhabitants of the Kat River valley, from where the species was originally described.

## 1 INTRODUCTION

Fishes of the cyprinid genus *Labeo* Cuvier, 1816 are widely distributed throughout the major river systems of Africa and Asia (Fricke et al., 2024). There are currently 114 valid species in this genus, with the highest diversity occurring on the African continent, where 77 species have been recorded (Fricke et al., 2024). The African *Labeo* species were divided by Reid (1985) into six species‐groups: *L. coubie* species‐group, *L. forskalii* species‐group, *L. gregorii* species‐group, *L. macrostoma* species‐group, *L. niloticus* species‐group and *L. umbratus* species‐group. New species from these groups are continuously being described, mainly from the Congo system and largely within the *L. forskalii* species‐group (Liyandja & Stiassny, 2023; Moritz, 2007; Moritz & Neumann, 2017; Tshibwabwa et al., 2006; Tshibwabwa & Teugels, 1995). However, a taxonomic revision of southern African labeos has received far less attention in recent years despite this region having a number of species with either disjunct or widespread distributions (see Skelton, 2001, 2024). These distribution patterns have often been found to obscure taxonomic diversity within a number of freshwater fishes (Kambikambi et al., 2021; Mazungula & Chakona, 2021; Mutizwa et al., 2021).

Southern Africa currently harbours 12 valid *Labeo* species (Fricke et al., 2024; Skelton, 2001). These species have been divided into the *L. umbratus* species‐group with four species [*L. umbratus* (Smith, 1841), *L. capensis* (Smith, 1841), *L. seeberi* Gilchrist & Thompson, 1913, and *L. rubromaculatus* Gilchrist & Thompson, 1913], the *L. forskalii* species‐group also with four species (*L. cylindricus* Peters, 1852, *L. molybdinus* Du Plessis, 1963, *L. lunatus* Jubb, 1963, and *L. ansorgii* Boulenger, 1907), the *L. niloticus* species‐group with three species (*L. altivelis* Peters, 1852, *L. rosae* Steindachner, 1894, and *L. ruddi* Boulenger, 1907) and the *L. coubie* species‐group with one species (*L. congoro* Peters, 1852). The phylogenetic relationships of these species are currently under study, with preliminary data supporting these divisions, although internal relationships are not well resolved (Ramoejane, 2016; Ramoejane et al., in preparation). The *Labeo umbratus* species‐group is the only group that is endemic to South Africa (Fricke et al., 2024; Skelton, 2001). All species within this group have narrow distribution ranges as each of them is confined to a single river system (Skelton, 2001). The only exception is *L. umbratus* (Smith, 1841), which shows a divided distribution: a northern population in the Orange‐Vaal River system and a southern population occurring in eight isolated, southward‐draining river systems, from the Gouritz in the west to the Nahoon in the east (Skelton, 2001).

In a recent phylogeographic study that incorporated multiple samples from across the distribution range of *L. umbratus*, this species was found to comprise two distinct lineages that are allopatrically distributed (Ramoejane et al., 2021). These were designated in that study as the northern (endemic to the Orange‐Vaal River system) and southern (distributed from the Gouritz to the Nahoon River systems) lineages (Ramoejane et al., 2021). The southern lineage exhibits considerable substructuring, comprising three regional haplogroups: (i) a western haplogroup in the Gouritz River system, (ii) a central haplogroup in the Gamtoos and Sundays rivers, and (iii) an eastern haplogroup extending from the Sundays to the Nahoon. These haplogroups display close relationships and shallow genealogical divergence, yet exhibit clear geographic localisation.

In the present study, we provide morphological evidence corroborating the distinctiveness of the eastern haplogroup from *L. umbratus s.s*. (i.e. the northern lineage in the Orange‐Vaal River system), supporting their recognition as distinct species. Smith (1841) described *L. umbratus* based on specimens that were collected from a tributary of the Vaal River in the Orange River system. An existing name corresponds to the eastern haplogroup of the southern lineage, as Castelnau (1861) described *L. cafer* from the Kat River (= Cat River), a major tributary of the Great Fish River system. According to the rules of the International Code of Zoological Nomenclature, this is the name that should be given to the eastern haplogroup as both genetic and morphological data support the revalidation of this species (see below). However, the species name is derogatory and highly offensive to the indigenous people of South Africa, where the species is endemic. The term ‘cafer’ is a corrupted form of the Arabic word ‘kafir’ which translates to ‘disbeliever’ or ‘non‐believer’. In the past, it was used as a derogatory term for non‐Muslims, particularly those of sub‐Saharan African descent who were enslaved. While the original meaning is religious, the term has been heavily racialised and is now considered offensive due to its historical usage.

While we acknowledge the importance of nomenclatural stability (Ceríaco et al., 2023), revalidating a name with such a harmful and offensive legacy contributes nothing to stability. This is because (i) retaining a derogatory epithet undermines the universality of zoological nomenclature by conflicting with ethical and cultural acceptance in the region of endemism, and (ii) the name has not been adopted in taxonomic literature since its proposal in 1861 and resurrecting it now would disrupt rather than promote nomenclatural stability. We therefore reject the use of *L. cafer* and instead provide a new name for the eastern haplogroup as a valid species. Accordingly, we present a comprehensive description of the eastern haplogroup of the southern lineage as a valid species under a new name, *Labeo ngqikai*, based on a detailed morphological assessment. By providing a formal and culturally appropriate name, we also establish an essential foundation for the conservation and management of this large, threatened cyprinid species.

## 2 MATERIALS AND METHODS

### 2.1 Ethics statement

The samples were collected using a combination of sampling gears, including seine nets, hand nets and electrofishing. Surveys for the freshly collected specimens followed the recommended ethics guidelines of NRF‐SAIAB (25/4/1/7/5_2022‐02). Research permits were obtained from the respective Provincial Conservation agencies: Free State (HK/P1/07871/001), Western Cape (AAA004‐00205‐0035), and Eastern Cape (HO/RSH/07/2023).

### 2.2 Study area and sample collection

Morphometric, meristic, and genetic data used in the present study were generated from fresh and historical specimens collected between 1977 and 2024 from a number of river systems encompassing the distribution range of *L. umbratus s.l*. The voucher specimens and DNA tissue samples are deposited in the National Fish Collection and National Aquatics BioBank at the NRF‐South African Institute for Aquatic Biodiversity (NRF‐SAIAB), Makhanda, South Africa. Fresh specimens of *Labeo ngqikai* were obtained from the Kat River, a tributary of the Great Fish River system, above the Kat River Dam in January 2024. This population represents the original stock of *Labeo* in the Great Fish River system prior to the translocation of the *L. umbratus* and *L. capensis* into the southward‐draining river systems following the construction of the Orange fish tunnel in 1975 (see Ramoejane et al., 2021). This is because the Kat River Dam was constructed in 1969 and thus prevented the upstream dispersal of the Orange River fish into the Upper Kat. A genetic study by Ramoejane et al. (2021) based on both mitochondrial Cyt b and nuclear S7 introns showed that the population of *Labeo* above Kat River Dam is genetically pure.

For freshly collected specimens, a subsample of the captured fish at each locality was anaesthetised with clove oil. A small piece of muscle tissue was dissected from the specimens and preserved in 95% ethanol. Source specimens were fixed in 10% formalin. On returning to the laboratory, DNA tissues were stored at −20 or −80°C and voucher specimens were transferred through 10% and 50%–70% ethanol for long‐term storage. The specimens were deposited into the National Fish Collection at NRF‐SAIAB in Makhanda (formerly Grahamstown), South Africa.

### 2.3 Molecular methods

Molecular analysis incorporated 171 archived Cyt b sequences of *Labeo umbratus s.l*. from Ramoejane et al. (2020, 2021) (Table 1). Six additional Cyt b sequences were generated for the newly collected specimens from the Kat River Dam (Table 1). Methods for obtaining Cyt b sequences followed Ramoejane et al. (2020). We designated sequences from the Vaal River as topogenetypes of *L. umbratus s.s*. (GenBank accession numbers: MF469467–MF469476) and those from the Kat River Dam as the topogenetypes for *L. ngqikai* (accession numbers: MF469505–MF469520, and PX361040–PX361045 for the six newly generated sequences) following Chakrabarty (2010). Archived Cyt b data for *L. capensis* (Orange River system at Parys and Kanoneiland) were obtained from Ramoejane et al. (2020) (GenBank accession numbers: MF469447–MF469466; Table 1). BioEdit 7.0.4 (Hall, 1999) was used to read and edit the DNA electropherograms of the newly generated sequences. The combined sequences were aligned with the ClustalW algorithm in MEGA 11 (Tamura et al., 2021). The substitution saturation was examined with DAMBE 7 (Xia, 2017). PopART 1.7 (Leigh & Bryant, 2015) was used to evaluate the phylogeographic patterns and to depict the evolutionary relationships among haplotypes within and between species based on the median‐joining method.

**TABLE 1:** Archived and newly generated Cyt b sequences of *Labeo ngqikai*, *L. umbratus s.s., L.* cf. *umbratus,* and *L. capensis* used in the present study.

Species	River system	Locality	Coordinates	*N*	GenBank accession no.	Reference
*L. ngqikai*	Great Fish	Kat River Dam	32°33′S, 26°46′E	6	PX361040–PX361045	This study
		Kat River Dam	32°33′S, 26°46′E	16	MF469505–MF469520	Ramoejane et al. (2020)
	Sundays	Slagboom Dam	33°22′S, 25°40′E	7	MF469488–MF469492 and MT904704–MT904705	Ramoejane et al. (2020), Ramoejane et al. (2021)
	Bushmans	Amakhala Game Reserve	33°31′S, 26°07′E	10	MF469495–MF469504	Ramoejane et al. (2020)
	Keiskamma	Middledrift Keiskammahoek	32°49′S, 26°59′E 32°41′S, 27°09′E	10 10	MF469521–MF469532 and MT904706–MT904713	Ramoejane et al. (2020), Ramoejane et al. (2021)
	Buffalo	Kwaklifu	32°56′S, 27°26′E	10	MT904714–MT904723	Ramoejane et al. (2021)
		Need's Camp	32°59′S, 27°38′E	10	MT904724–MT904733	Ramoejane et al. (2021)
	Nahoon	Nahoon Dam	32°54′S, 27°48′E	18	MT904734–MT904751	Ramoejane et al. (2021)
*L. umbratus s.s*.	Orange	Vaal Dam	26°51′S, 28°10′E	10	MF469467–MF469476	Ramoejane et al. (2020)
		Brak River	31°32′S, 22°20′E	10	MF469477–MF469486	Ramoejane et al. (2020)
		Gariep Dam	30°38′S, 25°33′E	28	MF469573–MF469575, MF469583–MF469596, MT904664–MT904674	Ramoejane et al. (2021)
*L*. cf. *umbratus*	Gouritz	Stompdrift Die Poort	33°30′S, 22°36′E 33°58′S, 21°39′E	5 5	MT904675–MT904684	Ramoejane et al. (2021)
	Gamtoos	Near Montpellier	33°13′S, 24°09′E	7	MT904685–MT904691	Ramoejane et al. (2021)
		Perdegat Pool	33°18′S, 24°20′E	12	MT904692–MT904703	Ramoejane et al. (2021)
	Sundays	Slagboom Dam	33°22′S, 25°40′E	3	MF469487, MF469493–MF469494	Ramoejane et al. (2020)
*L. capensis*	Orange	Parys	26°53′S, 27°27′E	10	MF469447–MF469456	Ramoejane et al. (2020)
		Kanoneiland	28°39′S, 21°05′E	10	MF469457–MF469466	Ramoejane et al. (2020)

Abbreviation: *N*, number of sequences.

### 2.4 Morphological methods

Methods used to obtain meristic and morphometric data followed Liyandja and Stiassny (2023), Ramoejane et al. (2020), Chakona and Swartz (2013), and Skelton (1988). Standard length (SL) is defined as the distance from the median anterior point of the snout to the base of the caudal fin, specifically the posterior end of the hypural plate. Additional morphometric characters were included to document the head and body morphometric variation: HwE, head width at eye; HwPrO, head width at preoperculum margin; HwO, maximum head width at operculum; HwJ, head width at mouth corner; PNL, anterior margin of nasal pore to tip of snout; HdNr, head depth at nasal pore; E‐J, distance from anterior margin of eye to corner of mouth; ULl, upper lip length, measured from mouth corner to tip of snout; HdE, head depth at eye; HdN, maximum head depth at nape; Chd, cheek depth; and Bd, maximum body depth in front of dorsal fin origin. Measurements were taken to the nearest 0.1 mm using dial callipers. Statistical analyses were carried out in IBM SPSS Statistics 27. Osteological analyses were carried out using an Inspex 20i Digital X‐ray Imaging System (Kodex Inc.) at NRF‐SAIAB. Morphological data for *Labeo rubromaculatus* and *L. seeberi* were taken from Reid (1985) and Skelton (2001, 2024), and supplemented by the authors' own observations.

## 3 RESULTS

### 3.1 Molecular data

The final Cyt b alignment (728 bp) included 177 sequences of *Labeo umbratus s.l*. and 20 sequences of *L. capensis*. The substitution saturation test of Xia et al. (2003) for all three codon positions showed that the Index of Substitution Saturation (Iss) was significantly lower (*p* < 0.0001) than the critical Index of Substitution Saturation (Iss.c) for symmetrical and asymmetrical topologies, indicating that the Cyt b sequences are applicable for genealogical analysis.

Sequence analysis of 197 specimens detected 25 variable nucleotide sites (seven singletons and 18 parsimony informative sites), leading to the definition of 23 haplotypes (H1–H23). The median‐joining haplotype network depicting genealogical relationships among these haplotypes revealed five distinct haplogroups with shallow divergence. A haplogroup corresponding to *L. capensis* (seven haplotypes, 20 individuals). A second haplogroup corresponding to the northern lineage of *L. umbratus s.l*. in Ramoejane et al. (2021), hereafter referred to as *L. umbratus s.s*., comprising all *L. umbratus* haplotypes from the Orange‐Vaal river system (three haplotypes, 48 individuals). The southern lineage of *L. umbratus s.l*. in Ramoejane et al. (2021) comprises three geographical haplogroups: (i) an eastern haplogroup, hereafter referred to as *Labeo ngqikai*, comprising all haplotypes from the Great Fish, Bushman's, Keiskamma, Buffalo and Nahoon river systems, as well as seven individuals from Sundays (seven haplotypes, 97 individuals); (ii) a western haplogroup, here referred to as *Labeo* cf. *umbratus*, comprising all haplotypes from the Gouritz River system (two haplotypes, 10 individuals); and (iii) a central haplogroup, also classified here as *Labeo* cf. *umbratus*, comprising all haplotypes from the Gamtoos River system as well as three individuals from Sundays (four haplotypes, 22 individuals).

**FIGURE 1:**
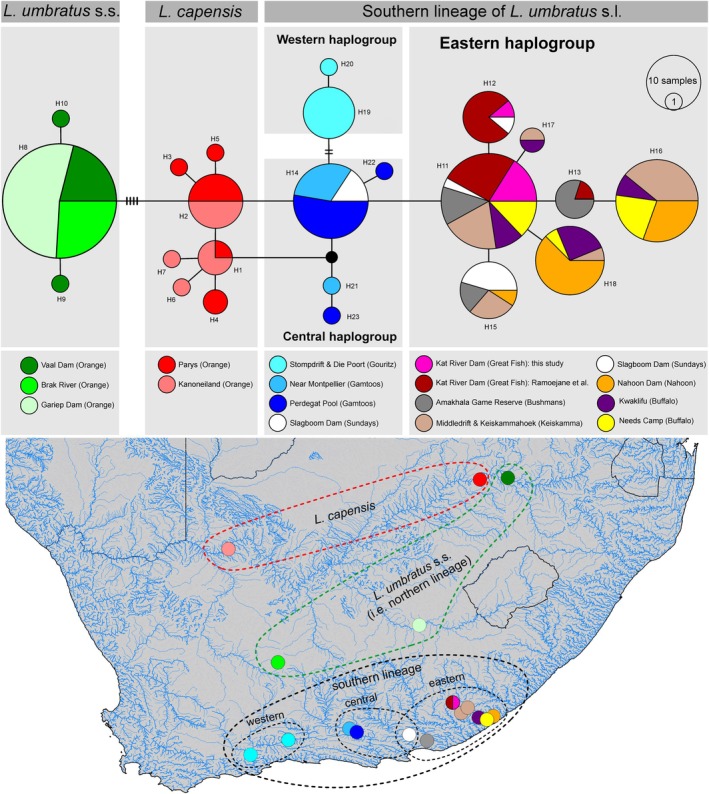
Median‐joining haplotype network indicating relationships among 23 Cyt b haplotypes (197 individuals) of putatively pure populations of *Labeo capensis* and *L. umbratus s.l*. from the Orange‐Vaal River system and the southern drainages of South Africa. Each line without hatch marks between two neighbouring haplotypes represents one mutational step. Circle sizes depict proportions of haplotypes; the smallest corresponds to one. A small black circle corresponds to a missing/hypothetical haplotype. Distributions of the species/haplogroups are depicted on the map.

The eastern, central and western haplogroups of the southern lineage, separated by one to three mutational steps in the sequence of Cyt b, form a monophyletic group in the haplotype network, more closely related to *L. capensis* than to *L. umbratus s.s*. from the Orange‐Vaal River system. *Labeo capensis* is separated from *L. umbratus s.s*. from the Orange‐Vaal River system by four mutational steps and from the southern lineage by one mutational step (Figure 1).

### 3.2 Morphological descriptions

Here, we present morphological evidence demonstrating the distinctiveness of the eastern haplogroup of the southern lineage from *Labeo umbratus s.s*. of the Orange–Vaal system, thereby supporting its recognition as a separate species. An epithet proposed in 1861 for this haplogroup, based on material from the Kat River, is inappropriate for continued use as it is both derogatory to South Africa's indigenous communities and absent from subsequent taxonomic usage. Reviving such an obsolete and offensive name would undermine, rather than strengthen, the universality and stability of zoological nomenclature. We therefore reject this historical epithet and designate the species under the new name *Labeo ngqikai*, under which we present a comprehensive description of the eastern haplogroup as a valid species, thereby promoting both nomenclatural stability and cultural sensitivity.

The central and western haplogroups of the southern lineage remain taxonomically unresolved as our preliminary morphological analysis of limited historical specimens from the Gouritz and Gamtoos suggests that these haplogroups do not correspond to *L. umbratus s.s*. or *L. ngqikai*. Future research should prioritise comprehensive integrative analyses that combine morphological and molecular approaches to investigate the central and western haplogroups of the southern lineage, utilising newly collected specimens and hybridisation analyses.

### 3.3 Taxonomic accounts

***Labeo ngqikai***

LSID urn:lsid:zoobank.org:act:5242C6C1‐9654‐43DF‐9FB0‐3696B4EC5A7E

Common name: Eastern Cape Labeo

**FIGURE 2:**
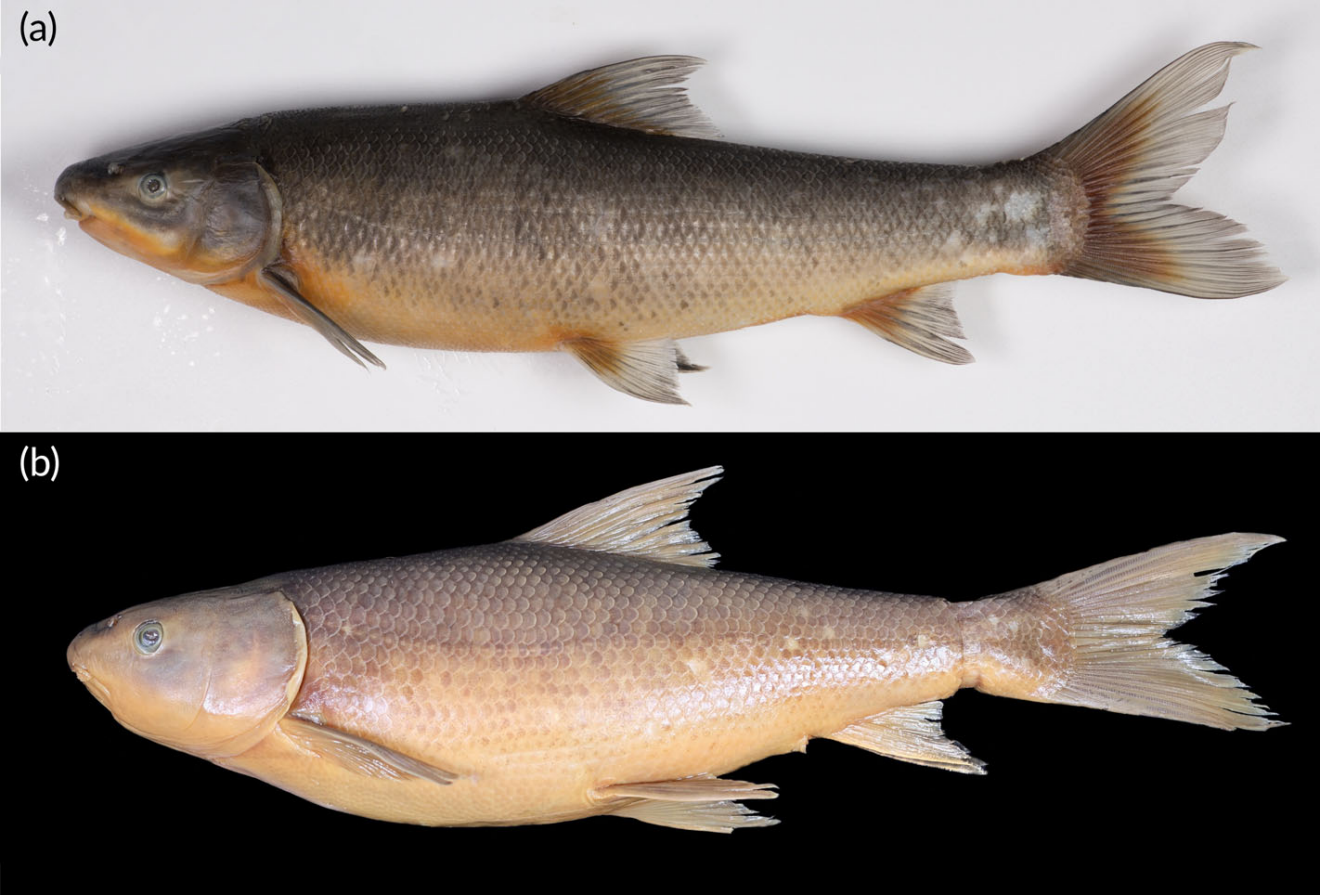
Lateral view of (a) *Labeo ngqikai*, SAIAB 246246, neotype, 262.2 mm standard length (SL), Kat River, and (b) *L. umbratus s.s*., SAIAB 188111 (tag no. MR08J025), 232 mm SL, Brak River, Orange‐Vaal system. Note the distinct differences in head and body shape and proportions between the two species (i.e. postorbital length, head depth at nares, cheek depth and maximum body depth in front of dorsal fin).

**FIGURE 3:**
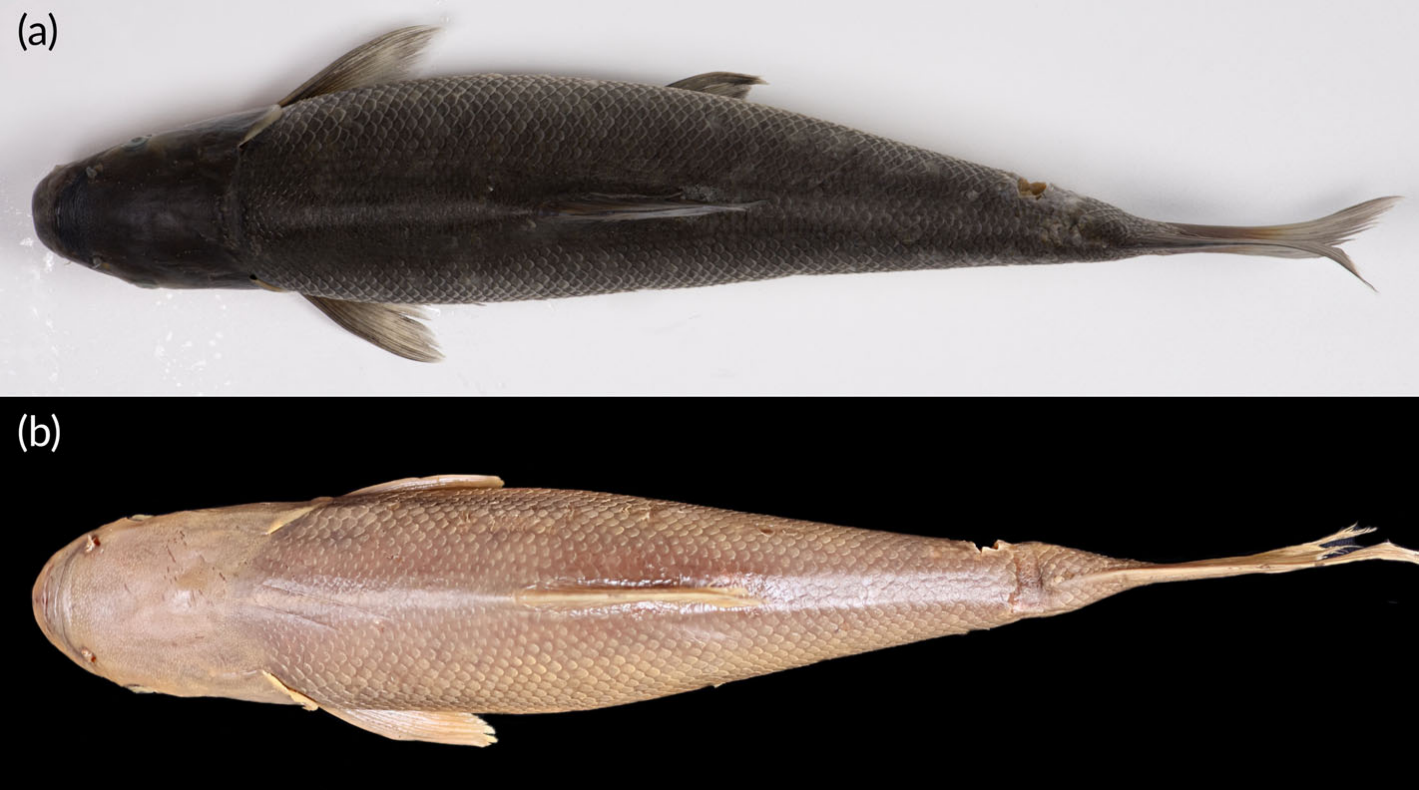
Dorsal view of (a) *Labeo ngqikai*, SAIAB 246246, neotype, 262.2 mm standard length (SL), Kat River, and (b) *L. umbratus* s.s., SAIAB 188111 (tag no. MR08J025), 232 mm SL, Brak River, Orange‐Vaal system. Note the distinct differences in head shape and proportions between the two species (e.g. distance between the nares, interorbital distance and head width at mouth angle).

**FIGURE 4:**
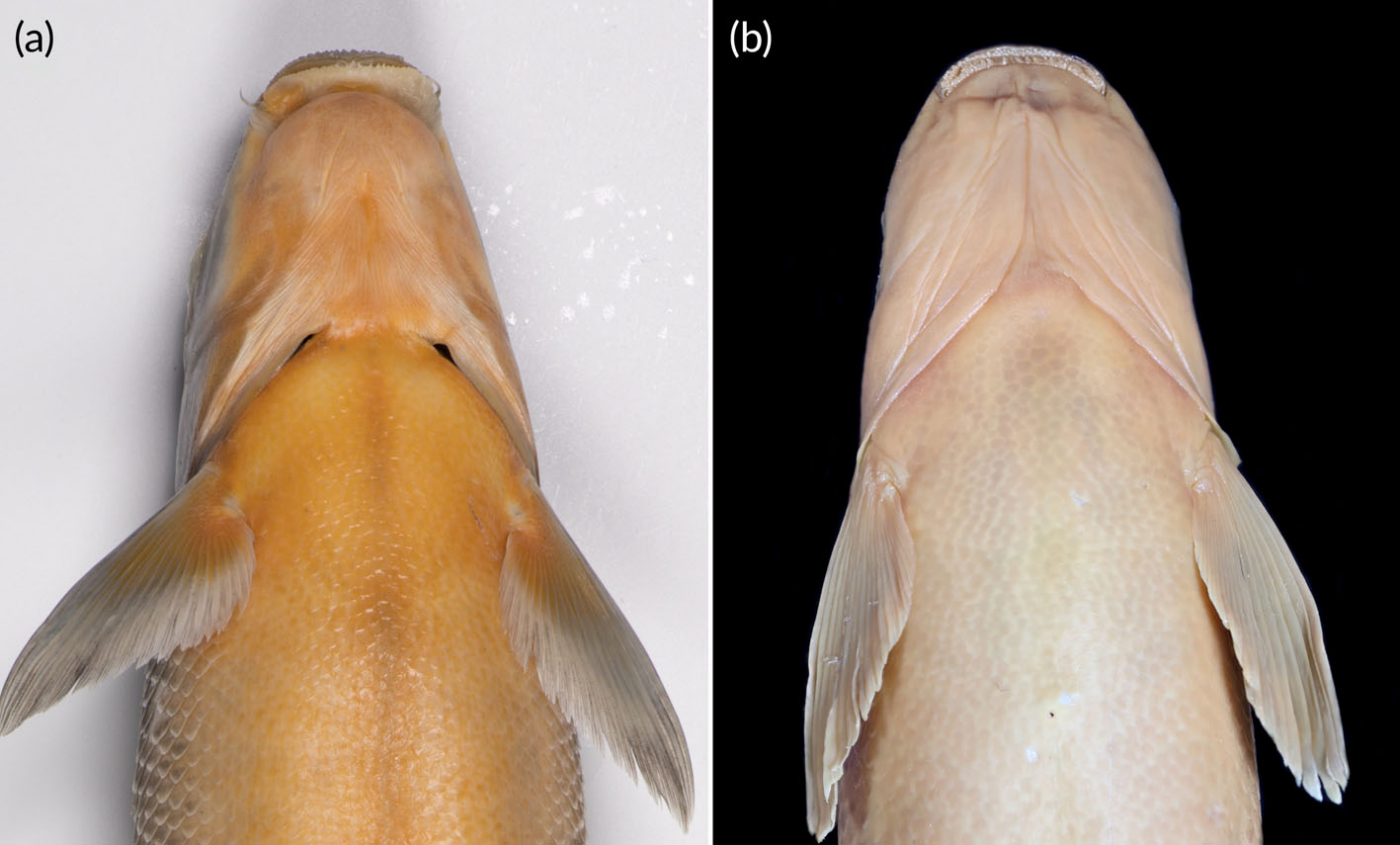
Ventral view of the head in (a) *Labeo ngqikai*, SAIAB 246246, neotype, 262.2 mm standard length (SL), Kat River, and (b) *L. umbratus s.s*., SAIAB 188111 (tag no. MR08J025), 232 mm SL, Brak River, Orange‐Vaal system. Note the distinct difference in head width at mouth corner between the two species.

**FIGURE 5:**
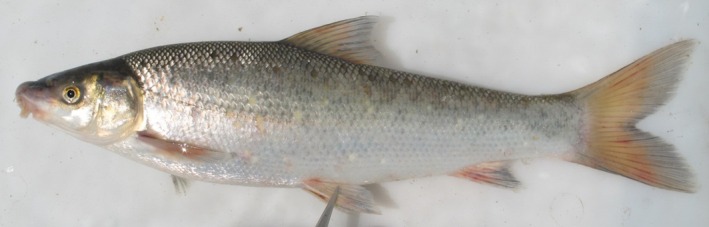
Freshly collected specimen of *Labeo ngqikai* from the Kat River. SAIAB 246078 (tag no. MR24‐A04), 185 mm standard length (SL).

Figures 2a, 3a, 4a and 5

*Labeo cafer* Castelnau, 1861:60 [Mémoire sur les poissons de l'Afrique australe], River crossing the Cat‐River‐Settlement, a branch of Great Fish River, northwestern Cafrerie, Cape Province, South Africa.

#### 3.3.1 Neotype

The type specimens originally used by Castelnau (1861) are currently untraceable. We therefore designate a neotype from the type locality, the Kat/Cat River, to provide a stable reference for *Labeo ngqikai*. SAIAB 246246 (tag no. MR24‐A01), unsexed, 262.2 mm SL, Kat River, Great Fish system, South Africa, −32.56, 26.77, collected by M. Ramoejane & M. Magoro, 24 January 2024. neogenetype: SB12337, GenBank number: PX361040.

#### 3.3.2 Additional material (*n* = 13)

SAIAB 246078 (tag no. MR24‐A02 to MR24‐A07), 6 unsexed, 167.3–244.7 mm SL, same locality information and collectors as SAIAB 246246 (Sequence ID: SB12338–SB12342, GenBank number: PX361041–PX361045). SAIAB 191165, 2 unsexed, 94.8–107.9 mm SL, Buffalo River, South Africa, −32.934, 27.44, collected by M. Ramoejane & R. Field, 26 October 2011. SAIAB 191164, 2 unsexed, 124.5–128.2 mm SL, Nahoon River, South Africa, −32.905, 27.809, collected by M. Ramoejane & R. Field, 26 October 2011. SAIAB 188023 (tag no. MR08G002 & MR08G015), 2 unsexed, 230.1–247.4 mm SL, Bushmans River, South Africa, −33.523, 26.124, collected by M. Ramoejane & B. Mackenzie, 4 November 2008. SAIAB 188033 (tag no. MR08G026), 1 unsexed, 245.3 mm SL, Bushmans River, South Africa, −33.517, 26.158, collected by M. Ramoejane & B. Mackenzie, 4 November 2008.

#### 3.3.3 Diagnosis

**FIGURE 6:**
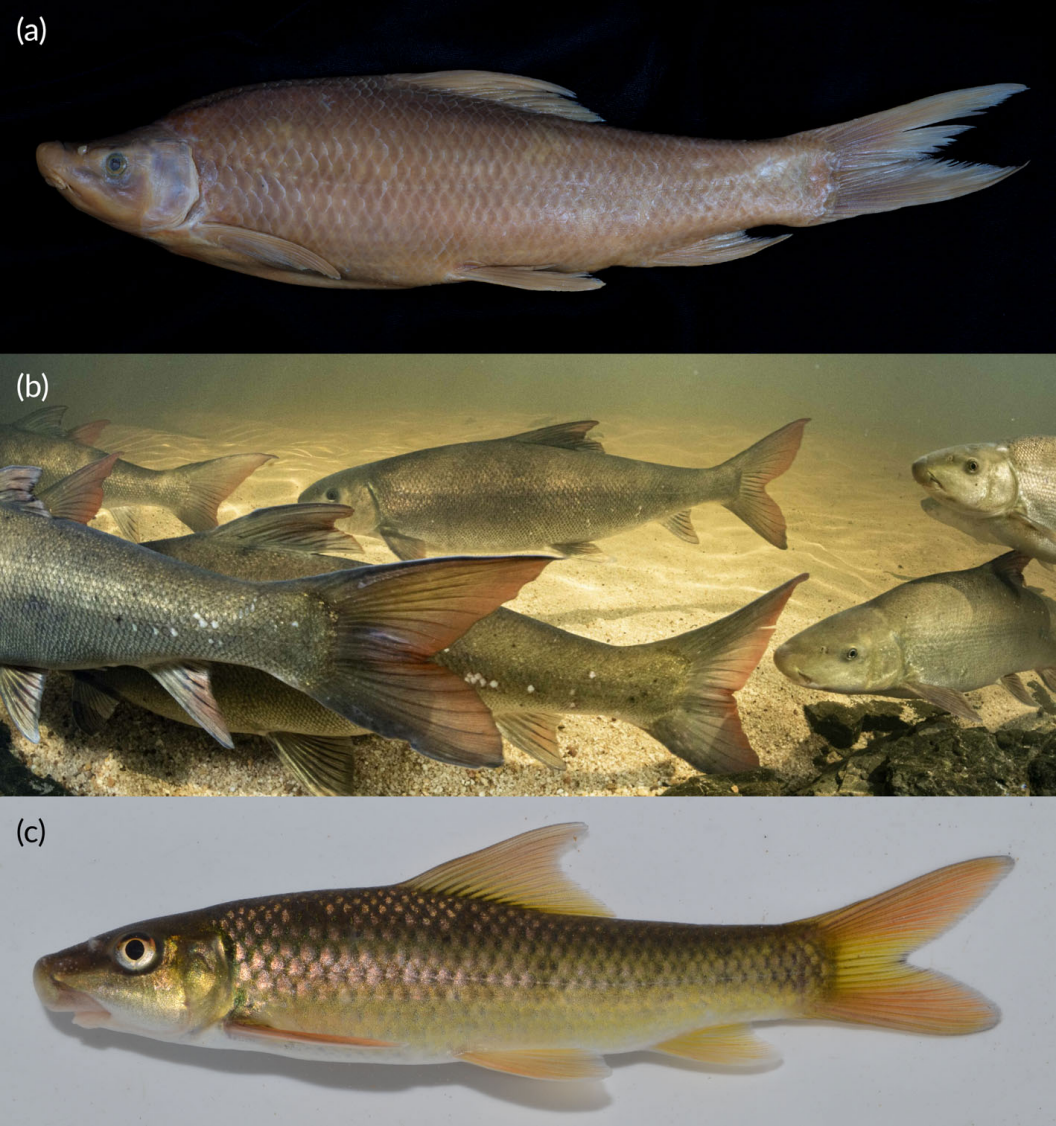
(a) *Labeo capensis*, SAIAB 187139 (tag no. MR08A051), 283 mm standard length, Orange River system, (b) *Labeo seeberi*, uncatalogued specimens, Biedouw River, a tributary of the Doring River (photograph by J. Shelton), and (c) *Labeo rubromaculatus*, SAIAB 210310, Tugela/Thukela River system.

A member of the *Labeo umbratus* species‐group based on Reid's (1985) definitions and clustering of the African labeos. *Labeo ngqikai* differs from *L. umbratus* s.s. (Orange‐Vaal system; Figures 2b, 3b and 4b) in having a narrower, shallower head and a shorter postorbital: interorbital distance 48.4–52.7 vs. 52.7–60.0% HL; head width at preoperculum 57.6–63.7 vs. 61.7%–67.8% HL; head width at operculum 53.1–68.9 vs. 67.5–73.4% HL; head width at mouth corner 34.2–41.6 vs. 42.1%–49.9% HL; left‐to‐right naris 28.2–32.2 vs. 32.2–36.2% HL; head depth at nares 39.1–46.9 vs. 44.8%–56.1% HL; cheek depth 20.7–31.3 vs. 30.5–34.1% HL; and postorbital length 42.0–46.0 vs. 48.2–53.4% HL. It further differs from *L. umbratus s.s*. in body and fin characters: maximum body depth 24.0–26.0 vs. 26.1–29.6% SL; dorsal fin base length 13.1–14.9 vs. 14.9–17.4% SL; predorsal scales 24–31 (usually 25–30) vs. 20–24 (usually 22–24); and pectoral‐fin branched rays 16–18 (usually 18) vs. 18–20 (usually 19). These species occur in historically isolated geographical systems. Compared with *L. capensis* (Orange‐Vaal system; Figure 6a), *L. ngqikai* has higher lateral‐line scales (59–66 vs. 44–46), circumpeduncular scales (29–33 vs. 22–25), predorsal scales (24–31 vs. 16–20), scales between the lateral line and dorsal‐fin origin (12–13 vs. 8–9), and scales between the lateral line and pelvic‐fin origin (9–10 vs. 6–7). It also has fewer dorsal‐fin rays (8–9 vs. 10–11), shorter dorsal‐fin length (18.4–22.0 vs. 25.9%–30.1% SL) and dorsal‐fin base (13.1–14.9 vs. 20.8%–22.4% SL), shorter anal‐fin length (12.7–15.8 vs. 21.1%–24.2% SL) and anal‐fin base (7.2–7.9 vs. 9.0%–10.3% SL), shorter pelvic fin (14.3–18.3 vs. 20.5–22.8% SL), and a straighter dorsal profile of the head (vs. concave in *L. capensis*; Figure 5 and 6a). Head proportions also differ: postorbital length 42.0–46.0 vs. 39.3%–40.9% HL, snout length 37.9–47.4 vs. 47.7%–50.7% HL, head width at mouth corner 34.2–41.6 vs. 44.2%–50.1% HL, and upper lip length 25.9–31.2 vs. 36.3%–38.4% HL. *Labeo ngqikai* differs from *L. seeberi* (Clanwilliam‐Olifants system; Figure 6b) in lateral‐line scales (59–66 vs. 75–90), circumpeduncular scales (29–33 vs. 36–50), scales between lateral line and dorsal‐fin origin (12–13 vs. 19–22), scales between lateral line and pelvic‐fin origin (9–10 vs. 15–18), head length (23.6–26.0 vs. 20.8%–23.8% SL), two pairs of barbels present (vs. anterior barbels absent in adults, posterior barbels reduced), total vertebrae (41–43 vs. 37–38), and precaudal vertebrae (27–30 vs. 23). *Labeo ngqikai* differs from *L. rubromaculatus* (Tugela/Thukela River system; Figure 6c) in lateral‐line scales (59–66 vs. 39–48), circumpeduncular scales (29–33 vs. 20–24), scales between lateral line and dorsal‐fin origin (12–13 vs. 8–10), scales between lateral line and pelvic‐fin origin (9–10 vs. 5–6), head length (23.6–26.0 vs. 20% SL), snout length (37.9–47.4 vs. 50% HL), interorbital distance (48.4–52.7 vs. 60% HL), eye diameter (12.1–21.2 vs. 7% HL), and absence of golden‐red flank coloration (vs. present in *L. rubromaculatus*).

#### 3.3.4 Description

The following description is based on 14 specimens from the Kat, Buffalo, Nahoon, and Bushman's rivers. All morphometric values in the text are presented as neotype first and other materials (13 specimens), if different, in parentheses.

#### 3.3.5 General morphology

Body proportions and meristics are given in Table 2. Body moderately elongate, fusiform, its depth in front of dorsal‐fin origin (deepest) 3.9 (3.8–4.1) in SL; body laterally compressed. Caudal peduncle shallow, its depth 0.6 (0.5–0.7) of caudal‐peduncle length. Head large, length 4.2 (4.0–4.2) in SL, depressed, depth at eye 7.4 (6.7–7.7) in SL and 0.5 (0.5–0.6) of maximum body depth. Postorbital profile steep. Snout moderately long, 2.1 (2.1–2.6) in head length. Eyes small, horizontal diameter 8.1 (4.7–8.3) in head length, and lateral, not extending above dorsal profile, located closer to tip of snout than posterior margin of operculum. Interorbital wide and slightly convex, 4.1 (2.4–4.1) of eye diameter. Mouth sub‐terminal with two pairs of short barbels.

**TABLE 2:** Morphometric and meristic data for *Labeo ngqikai* and *L. umbratus s.s*.

	*L. ngqikai*		*L. umbratus s.s*.
Character	Neotype	Range (*n* = 14^a^)	Range (*n* = 9)
Standard length (SL)	262.2	94.8–262.2	204.6–365.7
Meristics			
Lateral‐line scales	62	59–66	56–64
Scales between lateral line and dorsal‐fin origin	12	12–13	11–13
Scales between lateral line and pelvic‐fin origin	10	9–10	9–11
Circumpeduncular scales	30	29–33	28–34
Predorsal scales	31	24–31	20–24
Dorsal‐fin branched rays	9	8–9	9–9
Caudal‐fin branched rays	17	17–17	17–17
Anal‐fin branched rays	5	5–5	5–5
Pectoral‐fin branched rays	17	16–18	18–20
Pelvic‐fin branched rays	9	8–9	9–9
% of SL			
Maximum body depth (at dorsal‐fin origin)	25.5	24.0–26.0	26.1–29.6
Dorsal‐fin length along leading edge	19.2	18.4–22.0	20.5–23.8
Dorsal‐fin base length	13.7	13.1–14.9	14.9–17.4
Caudal‐peduncle depth	11.2	9.6–11.6	9.5–10.9
Posterior of dorsal fin to dorsal base of caudal fin	38.3	36.4–40.0	35.2–39.7
Pectoral‐fin length	18.6	15.3–20.3	19.1–23.0
Pelvic‐fin length along leading edge	15.9	14.3–18.3	17.8–23.3
Pectoral‐fin anterior origin to pelvic‐fin origin	31.0	28.3–31.6	28.6–31.6
Anal‐fin length along leading edge	15.0	12.7–15.8	15.0–17.3
Anal‐fin base length	7.8	7.2–7.9	6.9–8.4
Pelvic‐fin origin to anal‐fin origin	28.3	24.9–28.4	23.1–26.9
Dorsal‐fin origin to pectoral‐fin origin	31.4	29.3–33.7	29.1–33.0
Posterior of dorsal fin to pectoral‐fin origin	40.8	38.3–43.8	40.3–44.6
Dorsal‐fin origin to pelvic‐fin origin	24.9	24.0–29.1	25.4–29.2
Posterior of dorsal fin to pelvic‐fin origin	23.0	22.0–26.1	23.7–26.3
Dorsal fin origin to anal‐fin origin	38.3	37.2–39.2	37.6–41.3
Posterior of dorsal fin to anal‐fin origin	26.9	25.1–27.1	23.3–26.8
Posterior of dorsal fin to posterior of anal fin	30.3	29.1–30.6	27.6–31.0
Pectoral‐fin origin to ventral base of caudal fin	78.3	75.1–78.3	74.3–78.3
Anal‐fin origin to dorsal base of caudal fin	25.2	23.9–26.8	22.7–26.1
Caudal‐peduncle length	17.9	14.9–19.2	16.4–19.4
Posterior of anal fin to dorsal base of caudal fin	18.6	13.9–20.0	16.1–19.5
Snout to pectoral‐fin origin	23.5	23.5–25.4	23.2–26.6
Snout to pelvic‐fin origin	53.0	51.7–55.0	52.2–56.5
Snout to posterior of dorsal fin	59.9	58.8–62.5	58.5–64.0
Snout to anterior of dorsal fin	47.0	45.6–49.3	45.1–47.5
Head length (HL)	23.7	23.6–26.0	23.6–27.7
% of HL			
Operculum to preoperculum	30.4	24.9–30.4	27.5–34.6
Operculum to eye (postorbital length)	46.0	42.0–46.0	48.2–53.4
Eye to tip of snout (snout length)	46.6	37.9–47.4	41.4–45.1
Horizontal eye diameter	12.4	12.1–21.2	10.3–14.7
Eye to anterior margin of naris	18.8	15.8–19.1	16.7–20.7
Eye to posterior edge of naris	11.6	7.7–12.0	10.4–13.3
Inter‐narial distance	31.2	28.2–32.2	32.2–36.2
Interorbital distance	50.5	48.4–52.7	52.7–60.0
Head width at eye	50.3	50.3–61.7	57.5–63.9
Head width at preoperculum margin	61.9	57.6–63.7	61.7–67.8
Maximum head width at operculum	67.0	53.1–68.9	67.5–73.4
Head width at mouth corner	39.2	34.2–41.6	42.1–49.9
Naris to tip of snout	32.8	27.8–32.8	24.7–30.0
Head depth at nares	45.3	39.1–46.9	44.8–56.1
Eye to mouth corner	22.8	18.1–22.9	21.1–24.3
Upper lip length	27.3	25.9–31.2	25.7–30.1
Head depth at eye	57.3	54.9–60.8	56.2–62.5
Maximum head depth	77.8	70.4–79.6	70.5–82.3
Cheek depth	30.1	20.7–31.3	30.5–34.1

^a^ Neotype, six topotypes, and seven additional specimens from other river systems.

#### 3.3.6 Scales

Lateral‐line scale counts 59–66 (neotype: 62; other materials: 59:1, 60:1, 61:1, 62:1, 63:4, 64:2, 65:2, 66:1); scale count between lateral line and dorsal‐fin origin 12–13 (neotype: 12; other materials: 12:7, 13:6); scale count between lateral line and pelvic‐fin origin 9–10 (neotype: 10; other materials: 9:9, 10:4), circumpeduncular scale count 29–33 (neotype: 30; other materials: 29:2, 30:1, 31:3, 32:1, 33:6). Predorsal scale count 24–31 (neotype: 31; other materials: 24:1, 25:3; 26:1; 27:4; 29:1; 30:2; 31:1). Nape naked. Scales on predorsal, breast and abdomen smaller than flank scales. All scales cycloid.

#### 3.3.7 Fins

Dorsal‐fin rays iv/8–9 (neotype: iv/9; other materials: iv/8:1, iv/9:12); anal‐fin rays iv/5; pectoral‐fin rays 16–18 (neotype: 17; other materials: 16:3, 17:3; 18:7); pelvic‐fin rays i/8–9 (neotype: i/9; additional materials: i/8:1, i/9:12); branched caudal‐fin rays 17. Dorsal‐fin profile triangular, fourth spine longest, first to last branched ray shorten progressively; dorsal fin situated almost in the centre of the body (excluding caudal fin), origin significantly in front of vertical through origin of pelvic fin, distal margin concave. Pectoral fins fan‐shaped, significantly short of vertical through origin of dorsal fin. Pelvic‐fin origin almost below middle of dorsal fin, tip of depressed pelvic fin significantly short of anterior origin of anal fin in both sexes. Anal fin distal margin concave, origin closer to caudal fin base than anterior base of pelvic fin. Caudal fin forked.

#### 3.3.8 Osteology (*n* = 6)

Based on six specimens from the Kat River. Vertebral column including four vertebrae of the Weberian apparatus and urostyle: total vertebrae 41–43 (neotype: 42; other materials: 41:2, 42:1, 43:2), predorsal vertebrae 11–12 (neotype: 12; other materials: 11:3, 12:2), precaudal vertebrae 27–30 (neotype: 30; other materials: 27:3, 28:2), and caudal vertebrae 12–15 (neotype: 12; other materials: 14:2, 15:3).

#### 3.3.9 Colour in life

Refer to Figure 5 for live coloration. The head and body silvery grey, with the dorsal surface darker than the lighter, pale grey to white ventral surface. Flanks display flecks or marbled spots of varying intensity. Fins with orange‐red pigmentation on proximal parts.

#### 3.3.10 Colour in preservative

After 4 months in alcohol, specimens show countershading: darker dorsally, lighter ventrally (Figures 2a, 3a and 4a). No mid‐lateral band on flanks; dark centres on some scales create a flecked/marbled pattern. Fins are pale grey with orange‐red at bases.

#### 3.3.11 Distribution, habitat and ecology

*Labeo ngqikai* is endemic to the Eastern Cape Province of South Africa, where it has a narrow distribution range from the Nahoon to the Sundays River system (Figure 1). It prefers slow or gently flowing water and is abundant in impoundments. The diet of *L. ngqikai* consists of detritus and algae (Potts & Khumalo, 2005). The species is said to thrive in eutrophic systems compared to oligotrophic systems. These conditions are found mostly in ponds and man‐made reservoirs/dams, where they occur in high densities. This is due to the abundance of algae, which promotes its fast growth and thus matures faster. It spawns successfully during early spring and summer when there is consistent rainfall (Potts et al., 2005). This species is potamodromous and migrates upstream during flooding (Potts et al., 2005).

#### 3.3.12 Etymology

The species epithet *ngqikai* honours King Ngqika, the prominent leader of the Rharhabe‐Xhosas, who were the first to settle in the Kat River valley in the early 1800s. They would have most likely encountered these majestic fishes and possibly utilised them as a food resource. Chief Ngqika established his great place in the Kat River valley, the type locality for this species.

### 3.4 Comparative materials

*Labeo umbratus s.s*. (Smith, 1841). SAIAB 188111, 9 unsexed, 204.6–365.7 mm SL, Brak River, Orange‐Vaal River system, South Africa, −31.54, 22.343, collected by M. Ramoejane, E. Swartz & A. Chakona, 26 November 2008.

*Labeo* cf. *umbratus*. SAIAB 123444, 3 unsexed, 116.2–132.2 mm SL, Jan Muller bridge, Herbertsdale‐Van Wyksdorp, Gouritz River system, South Africa, −33.908, 21.653, collected by M. Curry, 10 March 1977. SAIAB 128465, 3 unsexed, 196.0–205.6 mm SL, Van Wyksdorp road, Causeway on Perdebont road, Gouritz River system, South Africa, −33.783, 21.735, collected by M. de Klerk & S. Thorne, 13 December 1982. SAIAB 188047, 12 unsexed, 170.7–340.5 mm SL, Perdegat pool near Steytlerville, Groot River, Gamtoos River system, South Africa, −33.311, 24.347, 5 December 2006.

*Labeo capensis* (Smith, 1841). SAIAB 187170 (tag no. MR08A016), 1 unsexed, 228.7 mm SL, Gariep E.C., Orange‐Vaal River system, South Africa, −30.64, 25.52, collected by M. Ramoejane, B. Ellender, D. Churches & H. Winker, 18 January 2008. SAIAB 187130 (tag no. MR08A023), 1 unsexed, 232.7 mm SL, Orange‐Vaal River system, South Africa, −30.639, 25.558, collected by B. Ellender, E. Swartz, D. Churches, M. Ramoejane, H. Winker, V. Mthombeni & B. Mackenzie, 11 January 2008. SAIAB 187135 (tag no. MR08A036), 1 unsexed, 299.6 mm SL, Orange River system, South Africa, −30.64, 25.774, collected by B. Ellender, D. Churches, M. Ramoejane & H. Winker, 12 January 2008. SAIAB 187139 (tag no. MR08A051), 1 unsexed, 283.0 mm SL, Orange River system, South Africa, −30.611, 25.789, collected by M. Ramoejane, B. Ellender, D. Churches & H. Winker, 13 January 2008. SAIAB 187145 (tag no. MR08A068), 1 unsexed, 273.7 mm SL, Orange River system, South Africa, −30.611, 25.789, collected by M. Ramoejane, B. Ellender, D. Churches & H. Winker, 13 January 2008.

## 4 DISCUSSION

### 4.1 Refining taxonomic diversity within *Labeo umbratus s.l*.

Ramoejane et al. (2020) found morphometric differentiation between the northern (Orange River system) and southern (southward‐flowing river systems) lineages of *Labeo umbratus s.l*. but no meristic differentiation, and suggested the need for further investigation. In this study, we presented morphological evidence supporting the distinctiveness of the eastern haplogroup of the southern lineage, described here as *Labeo ngqikai*, occurring from the Nahoon to the Sundays River system. This distinguishes it from *L. umbratus s.s*. of the Orange‐Vaal River system and substantiates their recognition as separate taxonomic entities. Future research on South African *Labeo* species should focus on an exhaustive integrative analysis, combining morphological and molecular data, to investigate the central and western haplogroups of the southern lineage from the Gamtoos and Gouritz river systems based on freshly collected specimens. This detailed approach is crucial for accurately determining their species‐level classification and refining their taxonomic status.

### 4.2 Phylogenetic relationships

Endemic to South Africa, the *Labeo umbratus* species‐group is characterised by a unique biogeographic pattern. While close phylogenetic relationships and minimal divergences among species and lineages within this group have been documented using mitochondrial Cyt b and nuclear S7 markers (Ramoejane et al., 2020, 2021, in preparation, and this study), the exact phylogenetic relationships among these species and their connections to the five other species groups proposed by Reid (1985) remain unclear, as noted by Ramoejane (2016). This ambiguity in intra‐ and inter‐group relationships is partly due to (i) limitations of the current phylogenetic markers, (ii) the occurrence of introgressive hybridisation and (iii) inadequate representation of Labeo species from these groups in the analyses. It is essential to expand genetic analyses with more suitable markers and enhance sampling efforts to resolve these issues and gain a more thorough understanding of their evolutionary connections.

The shallow genetic divergence between *Labeo capensis* and the species and haplogroups within the *Labeo umbratus* complex is not unexpected, as similar patterns have been observed among other species of the genus *Labeo*, such as the *Labeo niloticus* group, including *L. horie* and *L. senegalensis* (Ramoejane, 2016, unpublished data). Comparable trends are also reported for other large cyprinids, including species of *Labeobarbus* (Beshera & Harris, 2014; Bloomer et al., 2007; Nagelkerke et al., 2015). These findings suggest that genetic differentiation within large cyprinid groups may often be relatively limited, reflecting either recent divergence or ongoing gene flow between closely related species.

### 4.3 Conservation implications

A careful and accurate taxonomic assessment of South Africa's ichthyofauna is a prerequisite for sketching effective conservation measures. The taxonomic revision of *Labeo umbratus s.l*. has important conservation implications. It establishes the recognition of at least two distinct species (*L. umbratus s.s*. and *Labeo ngqikai*) that should be prioritised for conservation efforts, as well as highlights that *Labeo ngqikai* has a more restricted distribution compared to *L. umbratus s.s*. The phylogeographic result facilitates the identification of conservation units, enabling more effective allocation of limited conservation resources. *Labeo umbratus s.s*., *Labeo ngqikai,* and the central and western haplogroups of the southern lineage encounter distinct threats due to differences in their habitat ranges and the levels of degradation within these areas. As a result, each group should be considered for specialised management strategies that reflect their unique conditions and challenges. These species and haplogroups are especially at risk due to their confinement to single management units with narrowly defined distribution ranges. Such a limited geographic scope makes them particularly susceptible to environmental changes, thereby increasing their risk of extinction. Therefore, if a species or haplogroup is overexploited or extirpated due to human activities or other factors, it cannot be restored by relocating individuals from other species or haplogroups. Comprehensive surveys are essential to assess the status of each species and haplogroup, and to identify suitable management interventions. This is particularly important given the region's exposure to various environmental pressures, such as invasive fish species, declining water quality, fragmentation by man‐made impoundments and weirs, excessive water abstraction, land use changes, and alterations to riverine habitats (Chakona et al., 2022). Ramoejane et al. (2020) demonstrated that inter‐basin water transfers between the Orange and Great Fish River systems, as well as between the Great Fish and Sundays River systems, facilitated hybridisation between *L. capensis* and *Labeo ngqikai* in invaded river sections.

## 5 CONCLUSION

*Labeo ngqikai* represents a distinct species, endemic to the Eastern Cape Province of South Africa. This recognition increases the known species within the *Labeo umbratus* species‐group from four to five, thereby refining our understanding of the complexities within this taxonomic assemblage. Nonetheless, this progress highlights an urgent need for more robust conservation strategies, as *L. ngqikai* faces significant risks of population decline and potential extinction, particularly due to ongoing hybridisation issues in the Great Fish and Sundays river systems. A rigorous and ongoing monitoring program is essential for tracking hybridisation trends and implementing targeted measures to mitigate its detrimental impacts on the Eastern Cape Labeo.

## AUTHOR CONTRIBUTIONS

M.R.: Conceptualization, methodology, data curation, investigation, writing – original draft, and writing – review and editing. F.Z.: Conceptualization, methodology, data curation, investigation, formal analysis, software, visualisation, writing – original draft, and writing – review and editing. X.M.: Data curation, visualisation, software, and writing – review and editing. A.C.: Conceptualization, data curation, supervision, resources, writing – original draft, and writing – review and editing.

## FUNDING INFORMATION

Funding for the field surveys and molecular analyses was provided by the National Research Foundation through the REFRESH project [FBIP‐211006643719].

## Data Availability

The data supporting the findings of this study are available in GenBank. Sequence data have been submitted under accession numbers PX361040–PX361045, MF469447–MF469532, MF469573–MF469575, MF469583–MF469596, and MT904664–MT904751.
